# Charge Transfer, Complexes Formation and Furan Fragmentation Induced by Collisions with Low-Energy Helium Cations

**DOI:** 10.3390/ijms20236022

**Published:** 2019-11-29

**Authors:** Tomasz J. Wasowicz, Marta Łabuda, Boguslaw Pranszke

**Affiliations:** 1 Department of Physics of Electronic Phenomena, Gdansk University of Technology, ul. G. Narutowicza 11/12, 80-233 Gdansk, Poland; 2 Department of Theoretical Physics and Quantum Information, Gdansk University of Technology, ul. G. Narutowicza 11/12, 80-233 Gdansk, Poland; 3 Gdynia Maritime University, ul. Morska 81-87, 81-225 Gdynia, Poland; fizbp@ug.edu.pl; 4 Institute of Experimental Physics, University of Gdansk, ul. Wita Stwosza 59, 80-952 Gdansk, Poland

**Keywords:** ion-induced collision, DNA damage, charge transfer, furan fragmentation

## Abstract

The present work focuses on unraveling the collisional processes leading to the fragmentation of the gas-phase furan molecules under the He^+^ and He^2+^ cations impact in the energy range 5–2000 eV. The presence of different mechanisms was identified by the analysis of the optical fragmentation spectra measured using the collision-induced emission spectroscopy (CIES) in conjunction with the *ab initio* calculations. The measurements of the fragmentation spectra of furan were performed at the different kinetic energies of both cations. In consequence, several excited products were identified by their luminescence. Among them, the emission of helium atoms excited to the 1*s*4*d* ^1^D_2_, ^3^D_1,2,3_ states was recorded. The structure of the furan molecule lacks an He atom. Therefore, observation of its emission lines is spectroscopic evidence of an impact reaction occurring via relocation of the electronic charge between interacting entities. Moreover, the recorded spectra revealed significant variations of relative band intensities of the products along with the change of the projectile charge and its velocity. In particular, at lower velocities of He^+^, the relative cross-sections of dissociation products have prominent resonance-like maxima. In order to elucidate the experimental results, the calculations have been performed by using a high level of quantum chemistry methods. The calculations showed that in both impact systems two collisional processes preceded fragmentation. The first one is an electron transfer from furan molecules to cations that leads to the neutralization and further excitation of the cations. The second mechanism starts from the formation of the He−C_4_H_4_O^+/2+^ temporary clusters before decomposition, and it is responsible for the appearance of the narrow resonances in the relative cross-section curves.

## 1. Introduction

Ion-molecule interactions occur in many natural phenomena. They are the fundamental processes for the generation and evolution of numerous gas-phase compounds in the interstellar medium and the atmospheres of planets [1]. According to astrobiology, they could have played a role in the appearance of primitive forms of life when the cosmic rays irradiated compounds, changing them into the prebiotic building blocks of life [2,3]. Ion-molecule collisions also define various aspects of human-made reactions exploited in engineering. For example, the knowledge of the fundamental features of etching and deposition processes induced by focused ion beams allows one to develop efficient ion beam processing and fabrication techniques [4]. Investigations on the kinematics of such kind of interactions in plasmas provide information about ion formation, ion temperature, plasma parameters in the plasma volume, ion energy distributions in plasma sheath and the influence of ions on the sheath [5,6].

However, the most intense studies have been recently performed to understand the modifications induced by ion collisions with living cells, particularly the DNA/RNA molecules [7,8,9,10]. This is because using ionic beams in medical cancer treatment seems to be one of the most efficient ways to damage the malignant cells by irradiating deep-seated tumors [11]. The lesions are caused by the primary ionizing beams, and the secondary particles formed within the track and are inflicted to the DNA/RNA by bond ruptures between the building blocks. The DNA/RNA building blocks can also undergo essential disintegration [10]. However, deoxyribose moiety is particularly vulnerable to photon and cation impact, as González-Magaña et al. showed in a recent study on the ionization and fragmentation of trapped protonated dGCAT oligonucleotides irradiated by energetic photons and C^q+^ (*q* = 1, 2..) ions [12]. It was also found that the cleavages of the bonds at deoxyribose molecules via inelastic electron interaction represent intermediate stages in producing strand breaks, which are the most critical form of the lesions in radiation damage to DNA [13]. Therefore, determination of the weakest bonds of the backbone molecules of the RNA and the DNA helix (deoxyribose sugar, nitrogen bases, and their analogs) triggered by impact with different kinds of ions is highly required. However, sometimes the particular component does not exist in the gas-phase in the same form as in the DNA helix. For instance, in the gas-phase, deoxyribose is in a pyranose form but in DNA it has a furanose structure [14]. Consequently, to study the interaction of ionizing beams with its furanose structure, different analogs are applied as corresponding targets. In this context, furan molecule, C_4_H_4_O, is often used as an elementary analog to deoxyribose [15]. Its furanose ring is also a building unit of many biologically active molecules such as chlorophyll and vitamin B12 [16]. It is also present as significant species in the pyrolysis of fossil fuels [17]. Thus ionization and fragmentation of this compound have been extensively studied by both experimental and theoretical approaches [18,19,20,21,22,23,24,25,26,27,28,29,30,31,32,33].

However, the literature concerning the ion-induced processes occurring in the gas-phase furan is very scarce. Excluding our preliminary communication on He^+^-induced fragmentation of furan molecules [30], the insight into the collisional mechanisms was only shed by the experimental investigations on the interactions with protons (H^+^) for incident energies below 1 keV [32]. It was shown [32] that impact processes were dominated by an electron transfer from furan molecules to H^+^ projectiles. As a result, the neutralization of protons occurred, and the production of the excited hydrogen atoms was observed [32]. However, higher excited states of those hydrogens were not effectively populated. It was argued that not only extremely high electric fields can alter the branching ratios and lifetimes of individual states [34,35,36,37] but also the residual electric fields [38] cause the Stark mixing of the states. The only expected electric fields that could contributed in the depopulation of the *n* = 6, 7, 8, … levels of hydrogen [32] were due to the space charge [38] of the proton beam produced by its movement in the earth’s magnetic field.

Apart from those studies, to the best of our knowledge, no experimental or theoretical data were reported earlier on helium ions impinged upon furan molecules. Helium is second the most common element in the universe [39,40,41]. In hadron therapies, on the other hand, cancer cells are mostly exposed on proton, and C^+^ beams [42,43,44], but new investigations are undertaken regarding the application of other ionic projectiles, including alpha particles [7,44,45,46]. Thus, He^+^ and He^2+^ cations seem to be the ideal candidates as the model objects in the studies of the ion-neutral molecule interactions occurring in those environments. Therefore, the present work focuses on disentangling the collisional processes leading to the fragmentation of the gas-phase furan molecules under the helium cations impact in the energy range from 5 to 2000 eV. The presence of different mechanisms was identified by analyzing the optical spectra, measured using the collision-induced emission spectroscopy (CIES) technique and by molecular calculations using the high level of quantum chemistry methods.

## 2. Results and Discussion

### 2.1. Optical Fragmentation Spectra

**Figure 1:**
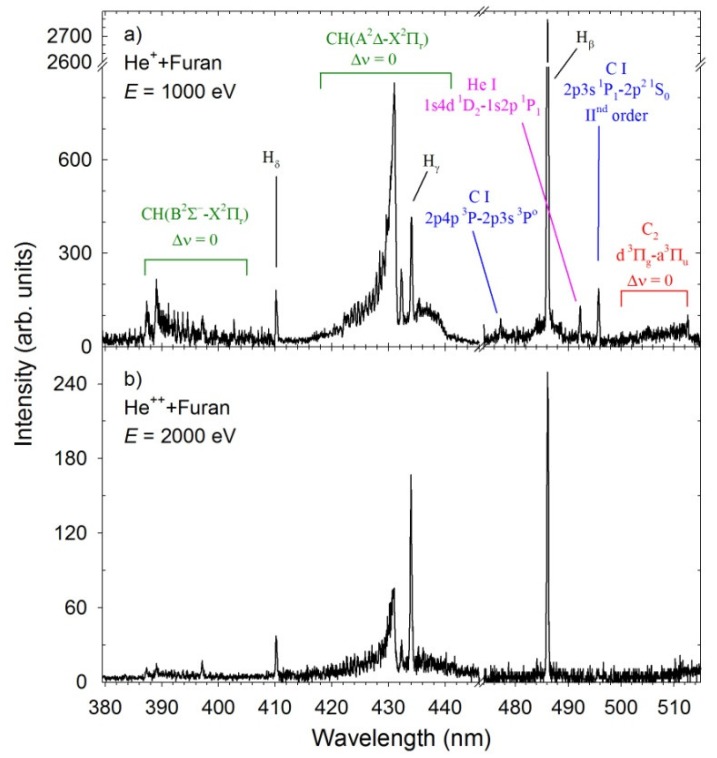
Luminescence spectra measured for collisions (**a**) He^+^ + furan, (**b**) He^2+^ + furan. The spectra were not corrected for the wavelength dependence of the sensitivity of the detection system. The green and red boxes represent ro-vibrational bands of the CH and C_2_ diatomic molecules, respectively.

In Figure 1 we present luminescence spectra measured for collisions between the He^+^ and He^2+^ cations and furan molecules. It is seen that the spectrum obtained in the He^+^ collisions (Figure 1a) is complex and contains luminescence from more fragmentation products than the spectrum recorded for the He^2+^ cations (Figure 1b). Both spectra contain the hydrogen lines of the Balmer series, H_β_ to H_ε_, due to the excited hydrogen H(*n*) atoms, *n* = 4−7, and the A^2^Δ→X^2^Π_r_ and B^2^Σ^+^→X^2^Π_r_ emission bands of the vibrationally and rotationally excited CH radical. However significant variations of relative intensities of these lines with changes of the projectile are seen. Moreover, the A^2^Δ→X^2^Π_r_ bands of CH molecule have different profiles. The luminescence spectrum measured for the He^+^ + C_4_H_4_O impact system shows additional products that were not detected in the He^2+^ + C_4_H_4_O collisions. It reveals the presence of the d^3^Π_g_→a^3^Π_u_ Swan system of the excited C_2_ fragments. Moreover, the carbon atomic species are identified as weak lines at ~477 and 495.7 nm. However, the most remarkable feature displayed in Figure 1a for the He^+^+furan collisions is the occurrence of emission from helium atoms excited to the 1*s*4*d* ^1^D_2_ state. The structure of the furan molecule lacks the He atom. Therefore observation of its emission lines is direct evidence of single electron transfer from target molecules to projectiles before the fragmentation.

**Figure 2:**
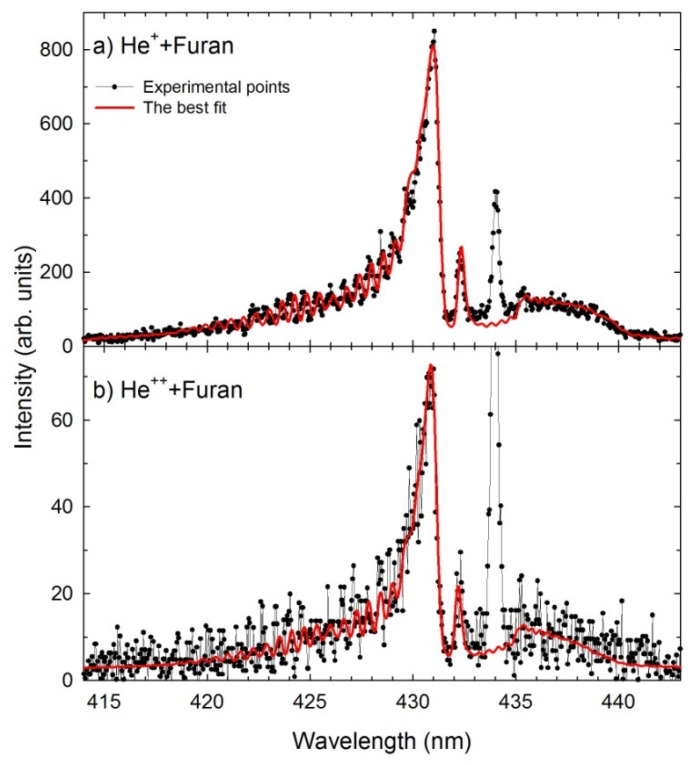
The experimental (black dots) and simulated (red contours) CH (A^2^Δ → X^2^Π_r_) spectra obtained in the (**a**) He^+^ + furan (*E* = 1000 eV), (**b**) He^2+^ + furan (*E* = 2000 eV).

To identify the differences in the CH(A^2^Δ→X^2^Π_r_), in each collision system, a theoretical spectrum was calculated utilizing the LIFBASE molecular spectra simulation program [47]^.^ In both cases, the CH(A^2^Δ→X^2^Π_r_) spectra were simulated with the use of vibrational and rotational constants of the A^2^Δ and X^2^Π_r_ electronic states of CH [48,49,50]. Moreover, the vibrational and rotational populations were assumed to be Boltzmann, governed by the characteristic vibrational (*T_v_*) and rotational (*T_r_*) temperatures. In both simulations, the Voigt profile for the apparatus function with a resolution *Δλ* of 0.39 nm (FWHM) was used. In Figure 2a,b, we have shown fittings performed for the collisions between the He^+^ + furan and He^2+^ + furan, respectively. 

The CH pattern found after the He^+^ impact was reproduced well for the vibrational and rotational temperatures of *T_v_* = 8500 K, *T_r_* = 4300 K (Figure 2a), respectively. The He^2+^ + furan collisions led to the creation of cooler CH fragment because the best agreement between simulated and measured spectra was achieved using the *T_v_* = 8000 K and *T_r_* = 3100 K (Figure 2b) temperatures. It is of note that the same computer procedure was employed in the analysis of the CH spectra occurring in the H^+^ + C_4_H_4_O [32] and C^+^/O^+^ + tetrahydrofuran (THF) [51] collisions. Not only did the proton irradiation yield a vibrationally and rotationally cold CH products similar to the He^2+^, but also a luminescence spectrum very similar to that recorded in the He^2+^ + furan collisions. The interactions with much heavier C^+^ and O^+^ cations led to the production of the CH products that were characterized by ~500 K higher temperatures than in the case of the He^+^ + furan collisions.

**Figure 3:**
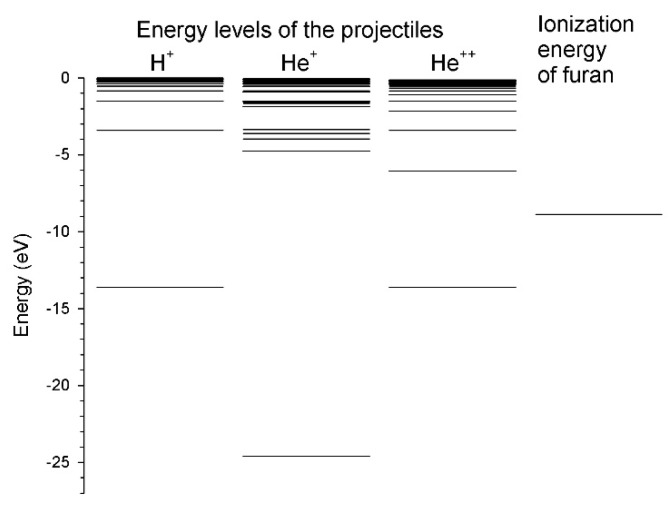
The energy levels of the H^+^, He^+^, and He^2+^ projectiles (taken from the National Institute of Standards and Technology (NIST) data collection [61]) in comparison with the ionization energy of furan (from [31]).

As we mentioned, the luminescence spectrum measured for collisions of He^2+^ cations with furan resembles the spectra obtained in the collisions of H^+^ + furan [32] and H^+^ + THF [51], respectively. The collisions with the H^+^/He^2+^ cations do not lead to efficient dissociation of the target molecules. This may suggest that the same collisional mechanism precedes dissociation and plays a crucial role in these reactions. A single electron transfer from the furan molecule to these projectiles may be such a possible process. Figure 3 presents the energy levels of the H^+^, He^+^, and He^2+^ projectiles in comparison with the ionization energy of furan. Both the target molecule and the H^+^/He^2+^ cations indeed have accessible states for the resonant electron capture processes. As seen in Figure 3, the resonant electron charge transfer from the furan molecule to the He^+^ is energetically precluded because of incongruity between the energy levels of these reactants. This may explain stronger fragmentation for He^+^ than He^2+^ or H^+^ where the resonant capture is possible. It is of note that the spectrum recorded in the He^+^ +furan collisions is similar to the fragmentation spectra of tetrahydrofuran bombarded by heavy hadrons (C^+^ and O^+^). Interaction with that massive cations creates conditions that enable many-body or even complete decomposition of target molecules [7,10,51,52]. We would like to point out that the results obtained in the electron- [27,53,54], and photon-induced [55,56,57,58,59] fragmentation of five- and six-membered heterocyclic molecules into neutral excited fragments always showed strong decomposition of those molecules. 

In contrast, the cation-induced dissociation mechanisms not only depend on the mass [32,51,52,60], but also the electron density of the projectile and therefore pave the way to selectively controlled reactions.

### 2.2. Relative Emission Cross-Sections

**Figure 4:**
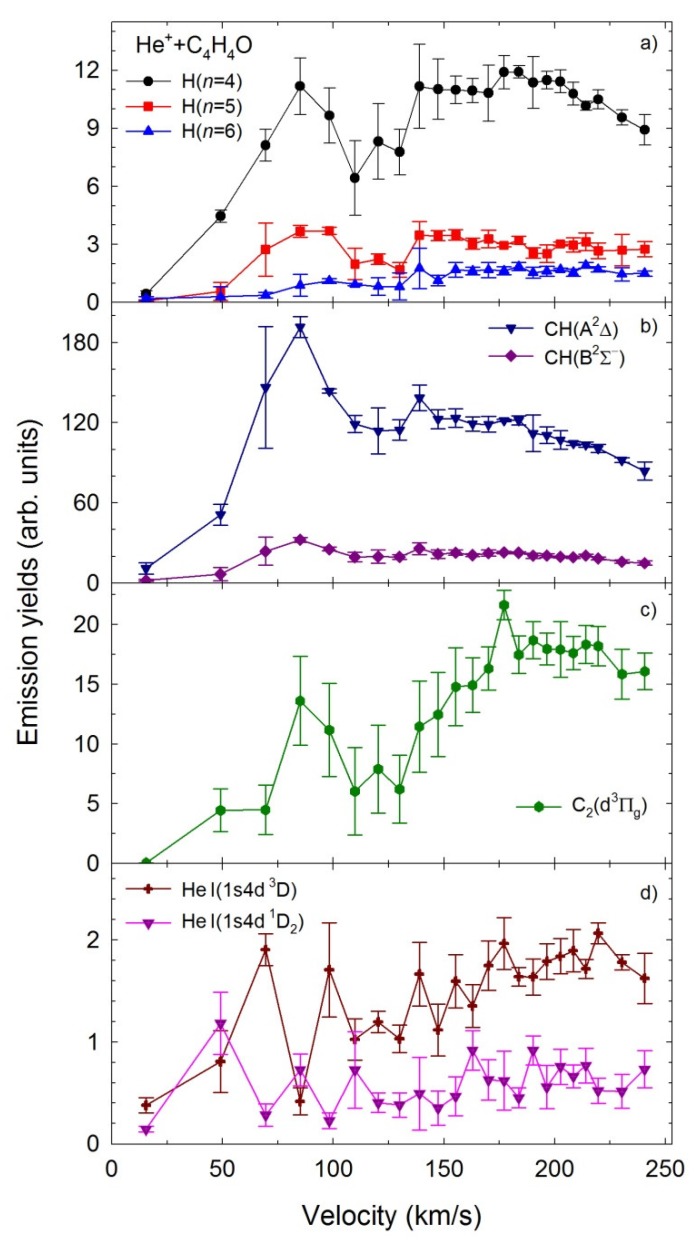
The emission yields of the excited products determined in collisions of He^+^ with furan molecules (**a**) H(*n*), *n* = 4–6, (**b**) CH (A^2^Δ, B^2^Σ^+^), (**c**) C_2_(d^3^Πg), (**d**) He I (1*s*4*d 3*D, ^1^D_2_).

The velocity dependence of He^+^ + furan relative emission cross-sections for the H(*n*), *n* = 4–6, CH (A^2^Δ, B^2^Σ^+^) and C_2_(d^3^Π_g_) fragments, and the most significant He I lines at 447.1, and 492.2 nm are presented in Figure 4. The experimental uncertainties in the emission yields are the mean standard deviations obtained from several independent measurements performed at fixed cation velocity. Due to the ion beam energy spread and thermal motion of the target [62], the uncertainty of collision velocity was estimated to be less than 1.5 km/s, and it is smaller than the size of the symbols.

It is seen that all of these curves have different contours that depend on the velocity. The H(*n*), CH (A^2^Δ, B^2^Σ^+^) and C_2_(d^3^Π_g_) curves rise rapidly above 15 km/s and show characteristic, narrow resonances with a maximum at 85 km/s and a half-width of ~50 km/s. Above 110 km/s, the emission yields of H(*n*) and C_2_(d^3^Π_g_) rise to a maximum at 175 km/s, and above 175 km/s, they start decreasing. The CH (A^2^Δ, B^2^Σ^+^) emission yields reach a plateau between 110 to 200 km/s, and then begin falling-off. Moreover, the CH(A^2^Δ) exhibits the largest relative emission cross-section.

The appearance of the narrow resonances in the H(*n*), CH(A^2^Δ, B^2^Σ^+^) and C_2_(d^3^Π_g_) cross sections seems to point at the formation of the ion-neutral molecule temporary complexes prior to dissociation [51,52]. Experimental and theoretical investigations show that such complexes are essential intermediates in many gas-phase reactions including collisions [51,52,63,64,65,66,67]. These studies showed that in collisional systems both interacting components are bound collectively by the ion-dipole electrostatic attraction. In the present experiment, the resonance velocities are quite low and the time of interaction of He cation and furan molecule is long enough to form the He−C_4_H_4_O^+^ temporary molecular cluster. Such a cluster is usually very unstable and it rapidly breaks up, leading to the formation of the observed fragments. The time of interaction between He^+^ and furan molecules becomes too short at higher velocities of the cations. Thus, both interacting components cannot bound together, and the production of the cluster starts becoming ineffective. In consequence, the emission yields decrease. It is of note that other collisional processes still occur, but they seem to be suppressed at the resonance velocities. Present observations are in agreement with the studies of the collisions of the C^+^ cations with tetrahydrofuran molecules [51], the hydrogenated derivatives of furan. At the emission yield of the CH (A^2^Δ) fragment, similar resonance was observed, which could not be explained as an effect of common abstraction or substitution reaction. Thus, the formation of a C−C_4_H_8_O^+^ short-lived complex prior decomposition was suggested due to the electrostatic attraction between the C^+^ cation and the THF dipole moment. This assumption was later proved theoretically by Erdmann et al. [63] who investigated the C^2+^ + THF collisions. The calculations of energy curves and corresponding couplings of the C^2+^ +THF system showed that strong delocalization of the electrons on the THF ring toward the C^2+^ projectile indeed opens up the real chemical bonding of the oxygen atom of THF and the cation, thus leading to the quasi-molecular complex formation. As will be shown in the next section, similar calculations have been performed by us to validate this scenario concerning He^q+^ + furan collisions.

In contrast, the excitation functions of helium lines do not show any resonances. Both curves tend to rise very slowly and show the lowest values in the relative cross-sections. This observation is understandable because of the collisional mechanism. In contrast to other fragments, the excitation and emission of helium atoms must be preceded by an electron transfer from furan molecules to He^+^ cations.

**Figure 5:**
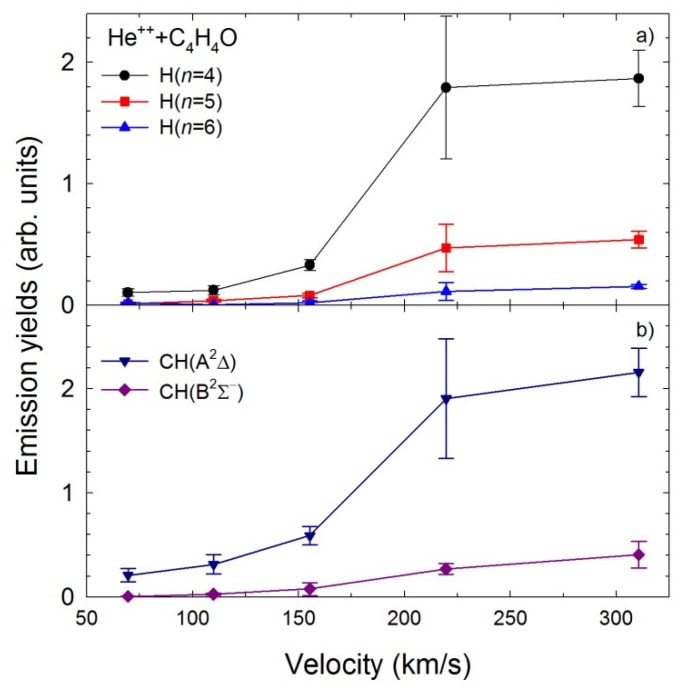
The emission yields of the excited products determined in collisions of He^2+^ with furan molecules (**a**) H(*n*), *n* = 4–6, (**b**) CH (A^2^Δ, B^2^Σ^+^).

The velocity dependence of the relative emission cross-sections obtained in the He^2+^ + C_4_H_4_O collisions are displayed in Figure 5a,b for the same fragments as shown in Figure 4a,b, respectively. However, different trends are observed for this collision system in comparison with the He^+^ + C_4_H_4_O impact. The emission yields show no resonances in the presented velocity range. Below the 110 km/s, all the relative cross-sections appear to be close to zero. Above 110 km/s, the H(*n*) and CH (A^2^Δ, B^2^Σ^+^) curves show a comparable increase as the velocity rises. Furthermore, as seen in Figure 4 and Figure 5, the Balmer series lines and the CH (A^2^Δ→X^2^Π_r_), CH (B^2^Σ^+^→X^2^Π_r_) bands are significantly more intense for the He^+^ + C_4_H_4_O system than the He^2+^ + C_4_H_4_O one.

### 2.3. Collisional Processes

It is difficult to ascertain unambiguously in such kind of experiments how the particular mechanism has proceeded. A definitive description can only be provided in conjunction with theory. Thus, the quantum chemical calculations were performed for both impact systems to get further insight into collisional processes.

From the theoretical point of view, the first step to investigate the electron exchange processes is a detailed analysis of the interactions occurring between molecular states involved in this process. Careful identification of the corresponding molecular configurations of the calculated states allows us to determine precisely not only the position of the possible electron ejection from the molecular target and its transfer towards the colliding ion but also to investigate the possibility of the formation of the quasi-molecular and ionized He−C_4_H_4_O^+/2+^ clusters.

Molecular results for the planar attack have been performed using C_2v_ symmetry group with consideration of the attack of the helium ions He^+^/He^2+^ along the He−O direction (*y* axis), corresponding to the *θ* = 0° angle. The molecular calculations for the collision of He^+/2+^ ions with the furan molecule have been investigated additionally in the perpendicular geometry. For this perpendicular collision, the colliding ion lying along the *x* axis and the furan molecule being in the *yz* plane with the origin of coordinates at the center of mass. The molecular system is considered in C_s_ symmetry.

**Figure 6:**
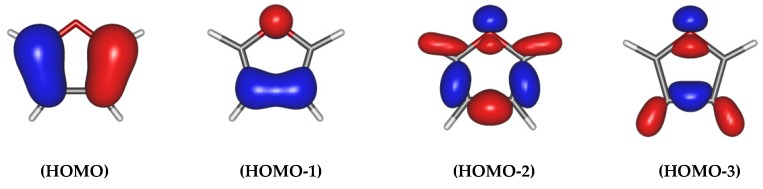
Main molecular orbitals involved in the charge transfer process located on the furan ring molecule. Orbital HOMO is defined as π_C1C2_(2p), HOMO-1 as π_C4C5_ (2p_x_) with some amount of o (2p_x_^O^), HOMO-2 as a π_C4C5_ (2p_y_)-indicated with the highest probability and HOMO-3 as (2p^O^).

In order to describe the interaction between an ion He^+^/He^2+^ and the furan molecule in both directions of the collision, only the highest occupied molecular orbitals (HOMO′s) have been taken into account (Figure 6) for the target molecule, the lowest orbitals being implicitly doubly occupied. A strong delocalization of the electrons is observed in furan. With this respect, we performed exact calculations to determine the energy levels, which are attributed to the main charge exchange channels. In all the presented cases, the chosen active spaces for the lowest states were constructed from an initial guess of Hartree-Fock orbitals by taking into consideration a combination of the (2p^O^) orbital, orbitals π_C4C5_ and π_C1C2_ on the heterocyclic molecule as well as 1s orbital on helium atom.

#### 2.3.1. He^+^ + C_4_H_4_O

**Figure 7:**
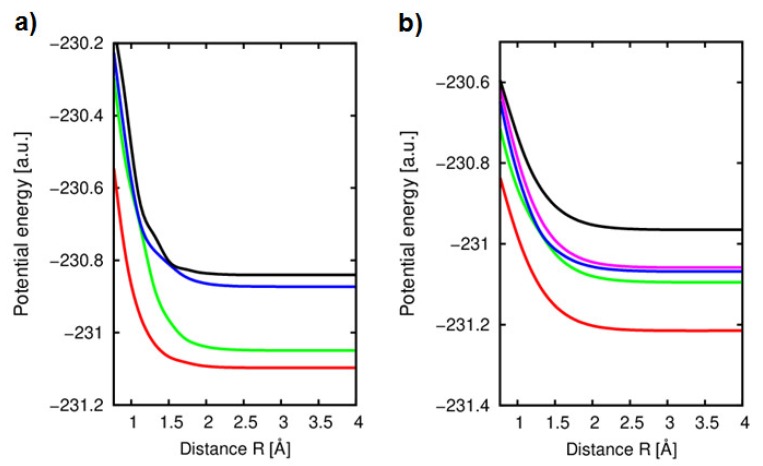
(**a**) Adiabatic potential energy curves of the ^2^A_1_ states in the collision of He^+^ ions with furan (C_2v_) in-the-plane orientation along the *y* axis. Numbering of states by increasing energy (color code online): 1 (red) corresponding to the configuration {n_O_(2p_x_^O^)^2^ (2p_z_^O^)^2^ π_C4C5_ (2p_x_)^2^ π_C1C2_ (2p_x_)^2^ π_C4C5_ (2p_y_) He (1s^2^)}; He−furan^+^; 2 (green) corresponding to {n_O_(2p_x_^O^)^2^ (2p_z_^O^) π_C4C5_ (2p_x_)^2^ π_C1C2_ (2p_x_)^2^ π_C4C5_ (2p_y_)^2^ He (1s^2^)}; He−furan^+^; 3 (blue) corresponding to {n_O_(2p_x_^O^)^2^ (2p_z_^O^)^2^ π_C4C5_ (2p_x_)^2^ π_C1C2_ (2p_x_)^2^ π_C4C5_ (2p_y_) He (1s^2^)}; He−furan^+^; 4 (black) corresponding to {n_O_(2p_x_^O^)^2^ (2p_z_^O^)^2^ π_C4C5_ (2p_x_)^2^ π_C1C2_ (2p_x_)^2^ π_C4C5_ (2p_y_)^2^ He^+^(1s)}; He^+^−furan. (**b**) Adiabatic potential energy curves of the ^1^A′ states in the collision of He^+^ ions with furan (C_s_) in the perpendicular orientation along the *x* axis. 1 (red) corresponding to the configuration {π_C1C2_ (2p_y_)^2^ (2p_x_^O^)^2^ (2p_z_^O^)^2^ π_C4C5_ (2p_y_)^2^ π_C4C5_ (2p_x_) π_C1C2_ (2p_x_)^2^ He(1s^2^)}; 2 (green) corresponding to the configuration {π_C1C2_ (2p_y_)^2^ (2p_x_^O^)^2^ (2p_z_^O^)^2^ π_C4C5_ (2p_y_) π_C4C5_ (2p_x_)^2^ π_C1C2_ (2p_x_)^2^ He(1s^2^)}; 3 (blue) corresponding to the configuration {π_C1C2_ (2p_y_)^2^ (2p_x_^O^) (2p_z_^O^)^2^ π_C4C5_ (2p_y_)^2^ π_C4C5_ (2p_x_)^2^ π_C1C2_ (2p_x_)^2^ He(1s^2^)}; 4 (magenta) corresponding to the configuration {π_C1C2_ (2p_y_)^2^ (2p_x_^O^)^2^ (2p_z_^O^) π_C4C5_ (2p_y_)^2^ π_C4C5_ (2p_x_)^2^ π_C1C2_ (2p_x_)^2^ He(1s^2^)}; 5 (black) corresponding to the configuration {π_C1C2_ (2p_y_)^2^ (2p_x_^O^)^2^ (2p_z_^O^)^2^ π_C4C5_ (2p_y_)^2^ π_C4C5_ (2p_x_)^2^ π_C1C2_ (2p_x_)^2^ He^+^(1s)}.

Firstly, in the molecular approach He^+^(1s) + C_4_H_4_O collision has been considered. This complex system is an open-shell system with orbital 1s being singly occupied on helium atom. For in-the-plane attack, the active space CAS (7,5) has been chosen. The potential energy curves for doublet states are illustrated in Figure 7a. An initial channel, attributed to the He^+^−C_4_H_4_O level corresponds to the {n_O_(2p_x_^O^)^2^ (2p_z_^O^)^2^ π_C4C5_ (2p_x_)^2^ π_C1C2_ (2p_x_)^2^ π_C4C5_ (2p_y_)^2^ He^+^(1s)} configuration.

In the immediate assessment of the results, we can observe that the curves are smooth and there is no direct charge transfer observed between the two lowest ^2^A states, corresponding to the He − C_4_H_4_O^+^ configuration and the entry channel 4^2^A. However, the strongest interaction seems to appear between the entry channel and 3^2^A which can be attributed to the {π_C4C5_ (2p_x_)^2^ π_C1C2_ (2p_x_)^2^ π_C4C5_ (2p_y_) He^+^(1s) level with the existence of the avoided crossing around *R* = 1.5 Å. A single electron capture takes place mostly from a π-one of the highest occupied molecular orbital of furan molecule, π_C4C5_ (2p_y_), to the singly occupied 1s orbital on helium. Moreover, more profound investigation of the evolution of the molecular configuration within the change of *R* showed a presence of the additional internal electron transfer in the ring of furan, which takes place from π_C4C5_ (2p_y_) orbital to the unoccupied π_C1C2_ (2p_x_) orbital. Indeed, the analysis of $\frac{\partial }{\partial R}$ radial coupling matrix elements with regard to the distance *R* between the considered states shows that only a smooth peak is observed for the 3^2^A–entry channel interaction in the neighborhood of the avoided crossing. Such interactions, although quite significant, seem however unlikely to form He − C_4_H_4_O^+^ with the great efficiency in He + C_4_H_4_O^+^ collision.

In order to have a more complete view on the mechanism of the electron transfer, the molecular calculations for the collision of He^+^ ion with furan have been performed in the perpendicular geometry of the collision attack. The calculated five potential energy curves for the case of He^+^ + C_4_H_4_O (C_s_ symmetry) collision process in perpendicular direction are displayed in Figure 7b. They show very interesting features and a quite different behavior may be pointed out with regard to in-the-plane molecular approach.

First of all, no direct interaction between the entry channel 5 ^2^A′ {π_C1C2_ (2p_y_)^2^ (2p_x_^O^)^2^ (2p_z_^O^) ^2^ π_C4C5_ (2p_y_)^2^ π_C4C5_ (2p_x_)^2^ π_C1C2_ (2p_x_)^2^ He^+^(1s)} and corresponding charge transfer levels are observed. Indeed, all of the curves exhibit very smooth trends, and they do not show any efficient avoided crossings and strong electron exchange between the considered doublet states. Detailed analysis of the evolution of the electronic configuration with respect to the internuclear distance *R* shows that the electron exchange is only observed in the ring molecule and correspond to the configurations of He − C_4_H_4_O^+^ (four lowest ^2^A′ states). Therefore, meaningful avoided crossing can only be pointed out between two ^2^A′ states (2 ^2^A′ and 3 ^2^A′) at the range of around *R* = 1.5 Å. It is confirmed by the analysis of the $\frac{\partial }{\partial R}$ radial coupling matrix elements with regard to the distance *R* between all of the states, which shows that only a smooth peak is observed for the g_23_ = $\langle \psi_{2}|\frac{\partial }{\partial R}|\psi_{3}\rangle$ coupling matrix element, reaching a maximum around 0.035 a.u. at a distance *R* = 1.3 Å. The other calculated couplings remain very small. Moreover, two states (3 ^2^A′ and 4 ^2^A′) are strongly degenerated and they correspond to the configurations which can be attributed to the He(1s^2^)−C_4_H_4_O^+^ levels, showing a single electron occupation on the molecular orbitals corresponding to the (2p_x_^O^) and (2p_z_^O^), respectively and a doubly occupied HOMO orbital π_C4C5_ (2p_x_)^2^.

#### 2.3.2. He^2+^ + C_4_H_4_O

**Figure 8:**
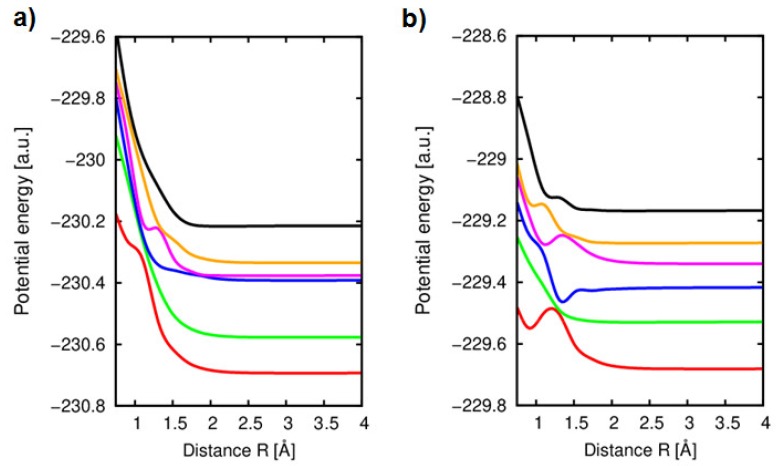
(**a**) Adiabatic potential energy curves of the ^1^A states in the collision of He^2+^ ions with furan in-the-plane orientation along the *x* axis. Numbering by increasing energy: 1 (red) corresponding to the configuration {n_O_(2p_x_^O^)^2^ π_C4C5_ (2p_x_)^2^ He(1s^2^)}; 2 (green) corresponding to the configuration {n_O_(2p_x_^O^)^2^ π_C1C2_ (2p_x_)^2^ He(1s^2^)}; 3 (blue) corresponding to the configuration {n_O_(2p_x_^O^)^2^ π_C4C5_ (2p_x_) π_C1C2_ (2p_x_) He(1s^2^)}; 4 (magenta) corresponding to the configuration {n_O_(2p_x_^O^)^2^ π_C4C5_ (2p_x_)^2^ π_C1C2_ (2p_x_) He^+^(1s)}; 5 (orange) corresponding to the configuration {n_O_(2p_x_^O^)^2^ π_C4C5_ (2p_x_) π_C1C2_ (2p_x_) He(1s^2^)};.6 (black) corresponding to the configuration {n_O_(2p_x_^O^)^2^ π_C4C5_ (2p_x_)^2^ π_C1C2_ (2p_x_)^2^ He^2+^}. (**b**) Adiabatic potential energy curves of the A′ states in the collision of He^2+^ ions with furan in the perpendicular orientation along the *x* axis. Numbering by increasing energy: 1 (red) corresponding to the configuration {n_O_(2p_x_^O^)^2^ π_C4C5_ (2p_y_)^2^ (2p_z_^O^)^2^ π_C4C5_ (2p_x_)^2^ He (1s^2^)}; 2 (green) corresponding to the configuration {n_O_(2p_x_^O^)^2^ π_C4C5_ (2p_y_)^2^ (2p_z_^O^)^2^ π_C4C5_ (2p_x_)^2^ π_C1C2_ (2p_x_)^2^ He(1s^2^)}; 3 (blue) corresponding to the configuration {n_O_(2p_x_^O^)^2^ π_C4C5_ (2p_y_)^2^ (2p_z_^O^)^2^ π_C4C5_ (2p_x_) π_C1C2_ (2p_x_)^2^ He^+^(1s)}; 4 (magenta) corresponding to the configuration {n_O_(2p_x_^O^)^2^ π_C4C5_ (2p_y_)^2^ (2p_z_^O^) π_C4C5_ (2p_x_) π_C1C2_ (2p_x_)^2^ He(1s^2^)}; 5 (orange) corresponding to the configuration {n_O_(2p_x_^O^)^2^ π_C4C5_ (2p_y_)^2^ (2p_z_^O^) π_C4C5_ (2p_x_)^2^ π_C1C2_ (2p_x_)^2^ He^+^(1s)}; 6 (black) corresponding to the configuration {n_O_(2p_x_^O^)^2^ π_C4C5_ (2p_y_)^2^ (2p_z_^O^)^2^ π_C4C5_ (2p_x_)^2^ π_C1C2_ (2p_x_)^2^ He^2+^}.

Similarly, the He^2+^(1*s*^2^) + furan closed-shell system has been considered, firstly in-the-plane symmetry. In this case, the lowest singlet states have been determined. For the He^2+^ + C_4_H_4_O, the calculations have been performed at the CASSCF level with an active space CAS (6,7) taking into account six electrons and seven orbitals located on the furan molecule including 1*s*^2^ orbital of the colliding helium atom which is involved in the process. In order to determine an exact configuration which corresponds to the He^2+^ + furan system, several state-averaged potential energy levels have been calculated. In Figure 8a, the main six adiabatic potential energy curves for the charge transfer levels of the He^2+^ + furan in-the-plane collision system are presented.

As it was expected, the lowest three ^1^A states are corresponding to the {n_O_(2p_x_^O^)^2^ π_C4C5_ (2p_x_)^2^ He(1s^2^)}, {n_O_(2p_x_^O^)^2^ π_C1C2_ (2p_x_)^2^ He(1s^2^)} and {n_O_(2p_x_^O^)^2^ π_C4C5_ (2p_x_) π_C1C2_ (2p_x_) He(1s^2^)} configurations, respectively, and no charge transfer is observed. They can be promptly attributed to the He(1s^2^) − C_4_H_4_O^2+^ level, where two electrons from furan are directly captured. Such a conclusion was confirmed by the determination of the non-adiabatic couplings, where the value of the corresponding coupling g_45_= $\langle \psi_{4}|\frac{\partial }{\partial R}|\psi_{5}\rangle$ =0.02 a.u. was indeed very small. Moreover, the molecular states 3^1^A and 4^1^A at asymptotic distance are corresponding to the same configuration {n_O_(2p_x_^O^)^2^ π_C4C5_ (2p_x_) π_C1C2_ (2p_x_) He(1s^2^)}, therefore one can conclude that at large enough distance of *R* they will be completely degenerated.

However, a significant avoided crossing around nuclear distance of 1.5–2 Å is clearly observed between the highly excited entry channel 5^1^A corresponding to {n_O_(2p_x_^O^)^2^ π_C4C5_ (2p_x_)^2^ π_C1C2_ (2p_x_)^2^ He^2+^} configuration and the charge transfer level 4^1^A of {n_O_(2p_x_^O^)^2^ π_C4C5_ (2p_x_) π_C1C2_ (2p_x_) He(1s^2^)}. It seems that with the decreasing of the distance between the two species, two electrons, one from orbital π_C4C5_ (2p_x_) and the second π_C1C2_ (2p_x_), which in the reference space are doubly occupied, were transferred to the He atom, fulfilling the lowest orbital 1s^2^, which missing the both electrons. The exact location of the transfer of those electrons is determined by the radial coupling g_45_, between the indicated states which gives the highest value. Analyzing the change of the wave function in the vicinity of the avoided crossing in the distance of around *R* = 1.5 Å we can indeed observe a formation of the temporary cluster, which is created when two electronic clouds of both the target and the projectile are starting to overlap, as it was reported in [63,68,69].

The potential energy curves for the He^2+^ + C_4_H_4_O in the perpendicular orientation are displayed in Figure 8b. In order to determine the entry channel, six states have been calculated in the chosen active space, CAS (6,6). As expected, the calculated states of the lowest energy correspond to the He(1s^2^) - C_4_H_4_O^2+^ or He^+^(1s)-C_4_H_4_O^+^ configurations, respectively, with the excitation of the electron from the highest occupied molecular orbitals of the furan molecule. Particularly the interactions between the states 1–3 and 4–5 show an interesting feature. They represent a furan molecule with a positive charge and a He cation with a single charge (states 3 and 5). It means, they represent a single-electron capture in a He^2+^ collision with the furan molecule, which is an entry channel. Due to the relatively short range exchange of the charge, the channels with existence of He^+^ show a coulomb repulsion and decrease as 1/R until they reach an asymptotic region. The calculated radial coupling matrix elements between these states, show very significant peaks in the vicinity of an avoided crossing, reaching maximum values of around 5 to 10 a.u at short range. For the R > 2.5 a.u., which is usually a region from which the separation of the both species is confirmed, the curves are flat and no avoided crossings leading to the charge exchange are observed. The entry channel is corresponding at long range to the configuration {n_O_(2p_x_^O^)^2^ π_C4C5_ (2p_y_)^2^ (2p_z_^O^)^2^ π_C4C5_ (2p_x_)^2^ π_C1C2_ (2p_x_)^2^ He^2+^} and it is strongly degenerated with the He^+^(1s)−C_4_H_4_O^+^ state corresponding to {n_O_(2p_x_^O^)^2^ π_C4C5_ (2p_y_) (2p_z_^O^)^2^ π_C4C5_ (2p_x_)^2^ π_C1C2_ (2p_x_)^2^ He^+^(1s)}. Indeed, a single electron transfer from π_C4C5_ (2p_y_) → He^+^(1s) orbital can be noticed at approximately *R* = 2 Å, showing the possibility of He−C_4_H_4_O^+^ complex formation in this collision. However, the most important feature with an avoided crossing at around *R* = 1.5 Å corresponding to a single excitation is exhibited between the entry channel and charge transfer level correlated asymptotically to He^+^−C_4_H_4_O^+^, {n_O_(2p_x_^O^)^2^ π_C4C5_ (2p_y_)^2^ (2p_z_^O^) π_C4C5_ (2p_x_)^2^ π_C1C2_ (2p_x_)^2^ He^+^(1s)} configuration.

Taking into account such a significant interactions observed, it seems that in the case of He^2+^+C_4_H_4_O collision, the double charge transfer mechanism is likely to occur via the formation of the temporary complex identified as a He - C_4_H_4_O^2+^. Nevertheless, this process remains somewhat mysterious at the moment because in our experiments we were not able to find any indication of its appearance. Undeniably we expected that this mechanism occurs and thus gives rise to excited He atoms just as in the case of He^+^+furan or He^q+^ + pyridine collisions [60]. However, apart from the H(*n*), CH (A^2^Δ, B^2^Σ^+^) emissions we did not detect any He nor He^+^ luminescence in the He^2+^ + furan collisions as seen in Figure 1.

### 2.4. Fragmentation Channels

It is very difficult to describe a complete mapping of the competing fragmentation pathways of furan molecule leading to atomic and diatomic fragments. Considering our results, it is immediately apparent that in the He^q+^ + C_4_H_4_O collisions possible dissociation channels leading to the production of the H, C, CH, and C_2_ fragments proceed through fragmentation of the parent cation of furan (C_4_H_4_O^+^) rather than a neutral molecule. Heterocyclic molecules usually decompose in double sequential dissociation reaction, where the primary dissociation products are subject to further decomposition. The C_4_H_4_O^+^ may thus dissociate into cations and corresponding neutral fragments. The charged products can be readily investigated using the mass spectrometric techniques, but the dissociation dynamics into neutral fragments is more challenging to study. However, if the mass spectrum unveils a high abundance of particular cation one can assume that corresponding neutral moiety is also efficiently generated. Those abundant neutral products may next decompose into smaller fragments such as detected in the present experiment. These small species should, therefore, coincide with the cations concentration in the furan mass spectra paving the way for plausible prediction of fragmentation pathways.

There is a limited number of experiments probing ion-furan reactions, and no direct comparison is possible to literature data. However, the studies on the electron-induced ionization and fragmentation of furan provided mass spectra [31] and exploited the opportunity to establish decomposition channels. The hydrogen fragments should be produced via the most straightforward fragmentation process, i.e., the direct abstraction of H atoms from the furan parent ion. However, a direct elimination of neutral H from C_4_H_4_O^+^ cation has a low probability, because the mass spectra showed a surprisingly low relative abundance (below 1%) of the C_4_H_3_O^+^ ion [31]. The newest theoretical investigation on neutral furan decomposition [33] showed that the skeleton fragmentation of furan has the lowest energy barrier, and it starts from the ring opening by cleavage of the weakest C−O bond followed by an isomerization and further breakage of the C−H, C−C, and/or C−O bonds. This scenario was indeed established in the experimental results. The most abundant cation in the mass spectrum of furan was assigned as C_3_H_3_^+^ (m/q = 39) [31]. The corresponding neutral moiety is the HCO molecule, which can split into the H and CO fragments. The abundances of the remaining cations were below 16% [31]. The only ones abundant enough were the cations with the masses of 42, 40, 38, and 29 amu. They were identified as C_2_H_2_O^+^, C_3_H_4_^+^, C_3_H_2_^+^, and HCO^+^, which were produced in the double, most likely sequential, dissociation of the parent furan cation leaving the C_2_H_2_, CO, H_2_CO, and C_3_H_3_ neutral species, respectively. These neutral moieties may then undergo multi-fragmentation yielding the H, C, CH, or C_2_ fragments. It is of note that all of these fragmentation pathways were recognized in calculations [33].

## 3. Materials and Methods

### 3.1. Experimental Method

The furan sample was purchased from Sigma Aldrich. A declared purity was better than 99%. It is liquid at room temperature (~20 °C), but it can be measured without heating the sample due to high vapour pressure (493 mm Hg) [70]. At the beginning of the experiment, the sample was degassed through several freeze-pump-thaw cycles to eliminate all traces of contaminating gases. It was found that the luminescence signal is a linear function of target gas pressure up to 30 mTorr, suggesting the single collision regime. Thus, during the investigation, the pressure of furan gas was kept constant at 15 mTorr, as determined with the Barocel capacitance manometer.

The experimental setup was described previously [71,72], but the most extensive characterization of this apparatus with its advantages and disadvantages was presented in the recent paper [32]. Moreover, the newest drawing of the equipment and methodology of measuring were shown in [51,52]. Briefly, the apparatus consisted of four parts: the ion source chamber, the magnetic mass selector, the collision chamber and the optical spectrometer. Both primary cations were created from the He gas utilizing an ion source of the Colutron hot cathode discharge type. The He gas was under a pressure of 0.75 Torr. The ions generated by cathode were extracted from the source using 1000 V voltage. Then, they were directed to a 60° magnetic mass selector, which separated them according to the m/q ratio. The carefully chosen cations were slowed down to the desired energy by immersion lenses. The ion beam energy varied from 5 to 1200 eV for the He^+^+furan reactions in this work, corresponding to the projectile’s velocities of about 15.5 to 240.6 km/s. Accordingly, the measurements with He^2+^ were performed in the energy range 100−2000 eV. These energies pertain to velocities of 69.4 to 310.6 km/s. The beam was then directed and focused into a small collision cell, where it impinged upon the vapors of the furan molecules. As a result of such collisions, some products in excited states occurred. They generated optical emission, which was recorded with a sensitive, multi-channel photon detector mounted in the optical spectrometer. The optical spectrometer operated on two gratings, namely a 1200 lines/mm grating and a 300 lines/mm one. The first grating allowed measuring the high-resolution optical fragmentation spectra (luminescence spectra) Δλ of 0.4 nm (FWHM), which is appropriate for the accurate identification of the spectral components. The second one allowed recording the luminescence signal with higher intensity, but a lower optical resolution (Δλ of 2.5 nm). We used this grating to record luminescence at a specific wavelength, as a function of the energy of incident cations. Each spectrum in this regime was corrected for the wavelength dependence of the sensitivity of the optical system. Then, the intensities of the emission lines were obtained by integrating over the peak/band areas. The background was taken to be the average of that below and above the studied lines and was subtracted from the spectra. Then, the intensities were normalized to the cation beam current and recording time. This provided the emission yield for the particular product., It represents the relative emission cross-section (σ) for the given reaction, leading to excitation of a product to a specific energy state. It is of note that the velocity dependence of emission yields (presented in this article) enables direct comparison of the relative emission cross-sections upon transition between various collision systems.

### 3.2. Theoretical Method

The following processes could have occurred during the He^+/2+^+ C_4_H_4_O collisions: (1) single or single/double electron transfer from the furan molecule to the He^+^ or He^2+^, respectively, and fragmentation of the furan cation; (2) dissociative ionization, which is the direct ionization of the furan molecule and further fragmentation of the furan cation; (3) dissociative excitation i.e., excitation of furan molecule and further fragmentation of the excited neutral molecule; (4) the transient cation-molecule complex formation prior to fragmentation.

In order to support the experimental investigations, the calculations have been performed by using quantum chemistry methods. Particular attention was paid to investigate the charge transfer process and possible formation of the quasi-molecular clusters between the colliding ions and target C_4_H_4_O molecule. Molecular description of the charge transfer process is based on the collision model at relatively low velocities. In this model, the nuclear motion is slow compared to the bound electron being captured. Under such conditions, the electron have sufficient time to adjust to the changing interatomic field as the nuclei approach and further time to separate. Thus, quasi-molecular description of the collision becomes necessary for a detailed treatment of the problem. The capture of electron process is represented as a transition between the stationary states of the quasi-molecule and depends strongly on the detailed analysis of the potential energy curves (PECs) representing the particular quasi-complex and on the couplings between them. Such treatment was firstly developed for the heavy ion-molecule polyatomic complex collisional systems (details in [68]), and later adapted to the series of the original applications, using mainly carbon ions as the projectiles [63,73,74,75] as well as few trials in protons. [76,77] In this model, detailed calculations for specific collision partners are performed utilizing the one-dimensional reaction coordinate approximation in which a projectile ion is approaching the corresponding target molecule by a single straight-line trajectory. Based on this assumption, the interaction of the heterocyclic C_4_H_4_O molecule with helium ions can be represented as the evolution of the polyatomic He−C_4_H_4_O^q+^ (*q* = 1, 2) quasi-molecular complex. In a first approximation, the complex can be treated as a pseudo-diatomic system with the reaction coordinate corresponding to the distance *R* between the centre of mass of the C_4_H_4_O molecule and the colliding ion. The rearrangement of the C_4_H_4_O molecule when approaching the He^+/2+^ ion can be taken into account by relaxation of the geometry of the He−C_4_H_4_O^q+^ complex along the chosen reaction path. Such an approach does not consider the degrees of freedom of the complex and the internal motions of the molecule but is quite reliable in a very fast collision process where nuclear vibration and rotation periods are assumed to be much longer (~10^−12^ s) than the collision time (typically 10^−16^ s).

**Figure 9:**
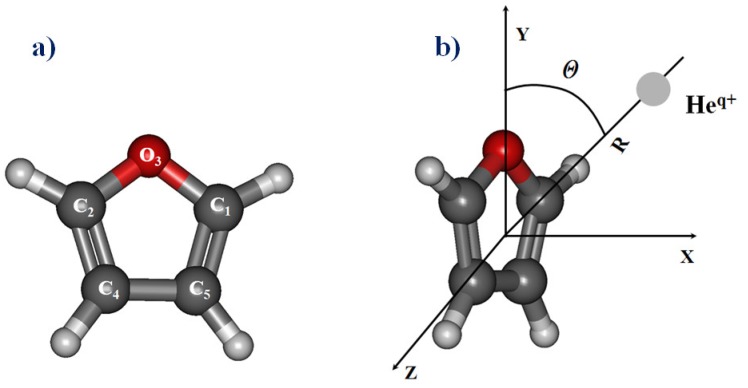
(**a**) Internal coordinates and numbering of the atoms in furan molecule; (**b**) Molecular model of the collision with He^q+^ cations: planar for *θ* = 0° and perpendicular *θ* = 90°.

Here, the geometry of the He^+^/He^2+^ + furan collision system is described using Cartesian coordinates with the origin at the centre of mass of the target molecule, as defined in Figure 9. The orientation of the projectile towards the molecular target may be studied for different values of the angle *θ*, from linear (*θ* = 0°) to perpendicular geometry (*θ* = 90°) taking into account of a high number of *R* distances in the range from 0.75 to 10 a.u. The choice of these two singular trajectories is representative for the collisions of ions with heterocyclic target molecules (as it was introduced in [68] and references therein), since they show the most different pattern for the potential energies and calculated cross-sections. There, the optimized structures and attachment sites have been calculated for multiply charges carbon ions complexes with uracil. These studies show for the different C ions a minimum energy for the carbon ion-uracil complex corresponding to an attachment site on oxygen which appears to be a preferential site.

The molecular calculations were carried out employing the MOLPRO package of *ab initio* programs [78,79]. The geometry of the ground state of the furan molecule has been optimized by means of density-functional-theory (DFT) calculations using the Becke-Lee-Yang-Parr density functional (B3LYP) [80,81,82] which has been shown to be computationally efficient and provides proper structures and transition energies [63].

Several tests have been performed, and the correlation-consistent aug-cc-pVQZ basis set of Dunning [83] has been chosen for colliding helium projectile and the 6–311++G ** for all of the remaining atoms in He^q+^ +furan systems. This choice was determined by the fact, that the larger basis set is applied, the fewer constrains on electrons are taken; therefore the exact molecular orbitals are more accurately approximated. However, the more precise parameters for the calculations are chosen, the more computational resources are required—especially with respect to the time of the calculations. The adiabatic PECs were computed at the state-average CASSCF (Complete Active Space Self Consistent Field) level of theory without consideration of the spin-orbit coupling. The most important molecular orbitals of the C_4_H_4_O molecule are chosen for the creation of the initial envisage for the active space. They are illustrated in Figure 6.

For all the cases considered, the default starting guess for the orbitals was taken from the Hartree-Fock orbital optimization. In order to compute several excited states, the energy average for all states under consideration have been optimized and a single set of the orbitals for all calculated states have been determined. In order to localize the charge transfer between the entry and corresponding exit channels, the non-adiabatic interactions should be taken into account, especially in the vicinity of the avoided crossings. A crucial part is to determine the position accurately and the height of the corresponding couplings, at least in two nearby internuclear distances, *R* and *R*+Δ, with Δ being very small. Therefore, for computing non-adiabatic coupling matrix elements (NACME′s) between the pairs of states of the same symmetry we use numerically efficient finite difference technique.

(1) $$g_{KL}^{rad}\left(R\right)=\langle \Psi_{K}|\frac{\partial }{\partial R}|\Psi_{L}\rangle =\lim_{\Delta \to 0}\frac{1}{\Delta }\langle \Psi_{K}(R)|\Psi_{L}(R+\Delta )-\Psi_{L}(R)\rangle \;=\underset{\Delta \to 0}{\lim}\frac{1}{\Delta }\left[\langle \Psi_{K}(R)|\Psi_{L}(R+\Delta )\rangle -\langle \Psi_{K}(R)|\Psi_{L}(R)\rangle \right]$$

where $|\Psi_{K}\rangle$ and $|\Psi_{L}\rangle$ are the eigenfunctions of the different molecular states involved in the process. The $\frac{\partial }{\partial R}$ operator is antisymetric and $\Psi_{K}$ and $\Psi_{L}$ are orthogonal, and then we obtain the final equation:

(2) $$g_{KL}^{rad}\left(R\right)=\underset{\Delta \to 0}{\lim}\frac{1}{\Delta }\langle \Psi_{K}(R)|\Psi_{L}(R+\Delta )\rangle$$

In order to assure numerical stability and accuracy of the two-point differentiation procedure, the Δ = 0.0012 a.u. have been chosen at *R* and *R*+Δ at every nuclear configuration. Such a value of Δ step has been previously tested for the calculation of couplings in several investigations of the charge exchange mechanism in simple [69,84,85,86,87] and more complex [63,68,73,74,75,76,77,88] collisional systems. Even though the CASSCF model has not been developed for treating dynamical correlation effects, it provides a good starting point for such studies, although the perturbation theory could be an alternative systematic approach to recover the correlation energy.

## 4. Conclusions

The present work provides novel data on low-energy helium cation collisions with furan molecule, an elementary model for the deoxyribose sugar unit. Collision-induced emission spectroscopy was applied to detect the luminescence of the excited products generated in the He^q+^ + C_4_H_4_O interactions. The following excited fragments were identified: atomic hydrogen H(*n*), *n* = 4–6, carbon atoms in the 2*p*3*s* ^1^P_1_, and 2*p*4*p* ^3^P states, and vibrationally and rotationally excited diatomic CH (A^2^Δ, B^2^Σ^+^) and C_2_(d^3^Π_g_) molecules. Moreover, the emissions of helium atoms excited to the 1*s*4*d* ^1^D_2_, ^3^D_1,2,3_ states were recorded. Emission yields of these excited products were measured as functions of the projectile energy/velocity. Products distribution and their relative intensities were different for both cations demonstrating that the fragmentation mechanism strongly depends on the charge of the cations. Thus, the role of different collisional processes was discussed. In particular, the charge transfer process and possible formation of the quasi-molecular clusters between the colliding ions and target C_4_H_4_O molecule were suggested.

In order to elucidate the experimental results, the complementary calculations were performed by means of quantum chemistry methods. From a theoretical point of view, this work provides accurate PECs and couplings for the collision of He^+^ and He^2+^ ions with furan molecule. In all the considered cases, the main channels which indicate purely existence of the He cations are high at energy and a lot of the states should be taken into account with the different symmetry configurations for each geometrical set up. It demands meticulous calculations with regard to the reference configurations between states. However, the calculated PECs in each case show an occurrence of avoided crossings around the nuclear distance of 1.5–2 Å. Such a region corresponds to the strong interaction between electronic states and exhibits the interplay between approaching electronic clouds of both species. Interestingly, we can determine the specific characterization of the molecular configurations in the very close-coupling region of the interaction, which is usually very difficult due to the strong interplay (many possible crossings) between the states. As a consequence it allows us to determine possible quasi-molecular clusters with He atom or its cations, especially, that most of the calculated couplings between the states appear to be rather smooth to lead to a very efficient reaction. We have in each case an excitation of the π electrons of the C=C double bonds of furan towards the 1s orbital on the helium ion projectiles. Our planar and perpendicular attack calculations show that the charge transfer depends strongly on the orientation of the molecule [68], projectile velocity [8,9] and its charge [8,9]. The calculated potential energy curves for the case of He^+^+ C_4_H_4_O collisions in both directions show that in-the-plane molecular approach there is stronger interaction between the entry and exit channels than in the perpendicular orientation. This leads to an efficient single electron capture. In case of the planar attack in the He^2+^ case, we could directly observe the double electron transfer from both π_C-C_ highest occupied orbitals on furan to the helium atom filling the electron gap. Moreover, the formation of the He−C_4_H_4_O^+/2+^ clusters seems more likely to occur close to the linear geometry than to perpendicular one.

A complementary study of the rotation of the molecule concerning the angle of attack of the ion should be useful in order to perform detailed orientation average calculations and average cross-sections.

These findings are qualitatively in agreement with recent experimental [30,32,51] and theoretical [63] studies on the interactions of different cations with furan and tetrahydrofuran molecules, respectively. The present results are also consistent with the most recent calculations of ion-induced charge transfer dynamics of isolated 2-deoxy-D-ribose [55] thus supporting the equivalence that was assumed between furanose and deoxyribose rings in the studies of their interactions with ions. Summarising, our results are promising, and they stimulated further analysis of the collision dynamics of the ion-biomolecule systems, which will be investigated in the incoming future.
